# Music listening while you learn: No influence of background music on verbal learning

**DOI:** 10.1186/1744-9081-6-3

**Published:** 2010-01-07

**Authors:** Lutz Jäncke, Pascale Sandmann

**Affiliations:** 1 University of Zurich, Psychological Institute, Department of Neuropsychology, Switzerland

## Abstract

**Background:**

Whether listening to background music enhances verbal learning performance is still disputed. In this study we investigated the influence of listening to background music on verbal learning performance and the associated brain activations.

**Methods:**

Musical excerpts were composed for this study to ensure that they were unknown to the subjects and designed to vary in tempo (fast vs. slow) and consonance (in-tune vs. out-of-tune). Noise was used as control stimulus. 75 subjects were randomly assigned to one of five groups and learned the presented verbal material (non-words with and without semantic connotation) with and without background music. Each group was exposed to one of five different background stimuli (in-tune fast, in-tune slow, out-of-tune fast, out-of-tune slow, and noise). As dependent variable, the number of learned words was used. In addition, event-related desynchronization (ERD) and event-related synchronization (ERS) of the EEG alpha-band were calculated as a measure for cortical activation.

**Results:**

We did not find any substantial and consistent influence of background music on verbal learning. There was neither an enhancement nor a decrease in verbal learning performance during the background stimulation conditions. We found however a stronger event-related desynchronization around 800 - 1200 ms after word presentation for the group exposed to in-tune fast music while they learned the verbal material. There was also a stronger event-related synchronization for the group exposed to out-of-tune fast music around 1600 - 2000 ms after word presentation.

**Conclusion:**

Verbal learning during the exposure to different background music varying in tempo and consonance did not influence learning of verbal material. There was neither an enhancing nor a detrimental effect on verbal learning performance. The EEG data suggest that the different acoustic background conditions evoke different cortical activations. The reason for these different cortical activations is unclear. The most plausible reason is that when background music draws more attention verbal learning performance is kept constant by the recruitment of compensatory mechanisms.

## Background

Whether background music influences performance in various tasks is a long-standing issue that has not yet been adequately addressed. Most published studies have concentrated on typical occupational tasks like office work, labour at the conveyer belt, or while driving a car [1-13]. These studies mainly concluded that background music has detrimental influences on the main task (here occupational tasks). However, the influence of background music was modulated by task complexity (the more complex the task the stronger was the detrimental effect of background music) [4], personality traits (with extraverts being more prone to be influenced by background music) [2,4-6], and mood [14]. In fact, these studies mostly emphasise that mood enhancement by pleasant and alerting background music enhances performance of monotonous tasks such as those during night shifts.

Whether background music influences performance of academic and school-related skills has also been investigated. A broad range of skills have been considered, including the impact of background music on learning mathematics, reading texts, solving problems, perceiving visual or auditory information, learning verbal material (vocabulary or poems), or during decision making [2,8,15-40]. The findings of these studies are mixed, but most of them revealed that background music exerts a detrimental influence on the primary academic task.

The present study was designed to readdress the question of whether background music enhances verbal learning. There are a number of reasons for this renewed interest: Firstly, only a few of the preceding studies have examined the effects of background music on verbal learning in particular [31,41-45], reporting more or less detrimental effects of background music on verbal learning, whereas several scientifically weak contributions have been published suggesting that listening to background music (in particular classical music) should have beneficial effects on learning languages [46,47]. Since verbal learning is an important part of academic achievement, we find it important to study the influence of background music on verbal learning more thoroughly. Secondly, the published studies used music of different genres (pop, classic) and vocals or they used instrumentals including musical pieces to elicit different emotions, music with different tempi, or simple tones as background music. Not one study has as yet controlled for the effects of emotion, complexity, tempo, and associated semantic knowledge of the musical pieces. The major aim of the present study was therefore to control these variables.

1. We used musical pieces unknown to the subjects. For this, we composed new musical pieces, avoiding any resemblance to well-known and familiar tunes. In doing this, we circumvented the well-known effect that particular contents of episodic and semantic memory are associated with musical pieces [48-50]. Thus, hearing a familiar musical piece might activate the episodic and semantic memory and lead to preferential/biased processing of the learned or the to be learned verbal stimuli.

2. A further step in avoiding activation of a semantic or episodic network was to use meaningless words. In combination with using unfamiliar musical pieces, this strategy ensures that established (or easy to establish) associations between musical pieces and particular words are not activated.

3. The musical pieces were designed in order to evoke pleasantness and activation to different degrees. Based on the mood-activation hypothesis proposed by Glenn Schellenberg and colleagues, we anticipated that music that evokes more pleasant affect might influence verbal learning more positively than music that evokes negative emotions [51-53].

4. Within the framework of the theory of *changing state effects *[54,55], we anticipated that rapidly changing auditory information would distract verbal learning more seriously than slowly changing music. Thus, slower musical pieces would exert less detrimental effects on verbal learning than faster music.

5. Given that the potentially beneficial effects of background music are also explained by a more or less unspecific cortical activation pattern, which should be evoked by the music and would change the activation of the cortical network involved in controlling learning and memory processes, we also registered EEG measures during learning and recognition. Here, we used event-related desynchronization and event-related synchronization of the EEG alpha band as indices of cortical activation. Our interest in event-related synchronization and desynchronization in the alpha band relates to the work of roughly the last 2 decades on alpha power demonstrating the relationship between alpha band power and cortical activity [56]. In addition, several recent combined EEG/fMRI and EEG/PET papers strongly indicate that power in the alpha-band is inversely related to activity in lateral frontal and parietal areas [57-59], and it has been shown that the alpha-band reflects cognitive and memory performance. For example, good memory performance is related to a large phasic (event-related) power decrease in the alpha-band [56].

## Methods

### Subjects

77 healthy volunteers took part at this experiment (38 men and 39 women). Two subjects were excluded because of data loss during the experiment. All subjects were recruited through advertisements placed at the University Zurich and ETH Zurich. All subjects underwent evaluation to screen for chronic diseases, mental disorders, medication, and drug or alcohol abuse. Normal hearing ability was confirmed for all subjects using standard audiometry. For intelligence assessment, a short test [60] was used that is known to correlate with standard intelligence test batteries (r = 0.7 - 0.8). In addition, the NEO-FFI [61] was used to measure the personality trait "extraversion" because of its strong correlation with dual task performance [4-6]. All subjects were tested for basic verbal learning ability using a standard German verbal learning test (Verbaler Lern- und Merkfähigkeitstest) [62,63]. All subjects were consistently right-handed, as assessed with the Annett-Handedness-Questionnaire [64]. All subjects indicated not having received formal musical education for more than five years during their school years and that they had not played any musical instrument in the last 5 years. We also asked the subjects whether they had previously learned while listening to music. Most of them conformed having done so, and a few (n = 5) indicated having done so frequently. The sample characteristics of the tested groups are listed in Table 1. There were no statistical between-group differences in these measures. Each subject gave written, informed consent and received 30 Swiss Francs for participation. The study was carried out in accordance with the Declaration of Helsinki principles and was approved by the ethics committee of the University of Zurich.

**Table 1:** Mean sample characteristics of the five groups studied.

	In-tune fast ITF	In-tune slow ITS	Out-of-tune fast OTF	Out-of-tune slow OTS	Noise
Age	23.8 ± 3.4	26.8 ± 4.9	27.3 ± 5.5	26.5 ±	25.3 ± 4.7
IQ	120 ± 9	118 ± 8	116 ± 16	119 ± 14	124 ± 5
Extraversion/introversion	2.6 ± 0.4	2.6 ± 0.4	2.4 ± 0.4	2.5 ± 0.5	2.6 ± 0.5
Years of education	15.6 ± 2.3	17.2 ± 3.7	16.1 ± 3.8	15.2 ± 2.0	14.6 ± 2.2
Gender n: f/m	8/7	8/7	8/7	8/7	7/8
N of subjects used to learn with music	4	2	1	2	5
Verbal memory (immediate recall)	45.5 ± 11.9	42.3 ± 11.5	41.7 ± 6.9	47.6 ± 10.5	45.4 ± 10.7
Verbal memory (delayed recognition)	17.3 ± 5.3	16.8 ± 4.3	16.7 ± 4.1	19.8 ± 3.9	17.2 ± 3.4

Indicated are means and standard deviations.

There was no statistically significant between-group difference for these measures (all p values at least >0.15).

### Study design

The basic principle of this study was to explore verbal memory performance under different acoustic background stimulation conditions. The subjects performed a verbal memory test (see below) while acoustic background stimuli were present (background+) or not present (background-). Four different musical pieces and a noise stimulus were used as acoustic background stimuli (in-tune fast, in-tune slow, out-of-tune fast, out-of-tune slow, noise; for a description of these acoustic stimuli see below). The 75 subjects were randomly assigned to one of these five groups, each group comprising therefore 15 subjects. These five groups did not differ in terms of age, IQ, or extraversion/introversion (tested with Kruskal-Wallis-U-test).

In the background+ condition, participants were required to learn while one of the above-mentioned background stimuli was present. Thus, the experiment comprised two factors: a grouping factor with five levels (Group: in-tune fast, in-tune slow, out-of-tune fast, out-of-tune slow, noise) and a repeated measurements factor with two levels (Background: without acoustic background [background-] and with acoustic background [background+]). We also measured the electroencephalogram (EEG) during the different verbal learning conditions to explore whether the different learning conditions are associated with particular cortical activation patterns. The order of background stimulation (verbal learning with or without background stimulation) was counterbalanced across all subjects. There was an intermittent period of 12-14 minutes between the two learning sessions during which the subjects rated the quality of the background stimuli and rested for approximately 8 minutes.

### Background stimuli

**Figure 1:**
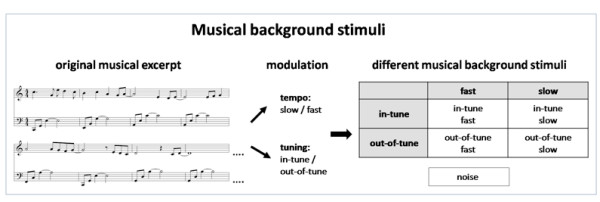
**Schematic description of the used musical excerpts (left panel).** On the right the experimental design is depicted.

Several studies have shown that tempo and the level of consonance/dissonance of musical excerpts strongly determine the level of arousal and emotional feelings [51,65,66]. We therefore designed 4 different 16 minute-long musical pieces differing in musical tempo and tuning. The musical excerpts were computerised piano sounds designed using FL Studio 4 software [67]. We composed a musical excerpt in C-major consisting of a melody and accompanying fundamental chords. This original musical excerpt was systematically varied in terms of tuning (in-tune, out-of-tune) and tempo (fast, slow), resulting in four different musical background stimuli (Figure 1). Two of these background stimuli were fast (in-tune fast, out-of-tune fast), and two of them were slow (in-tune slow, out-of-tune slow) (musical excerpts can be downloaded as supplementary material [68]). The in-tune excerpts comprised the typical semitone steps between the tones while in the out-of-tune excerpts the melody was pitch-shifted by one quarter-tone above the original pitch, resulting in the experience of out-of-tune. The tempo of the musical excerpts was varied by changing the beats per minute (160 bpm for fast and 60 bpm for slow) [51]. In addition, we designed a noise stimulus (also 16 minutes long; brown noise) with a temporal envelope similar to that of the other four musical excerpts. In summary, we applied five different kinds of background stimuli: in-tune fast, in-tune slow, out-of-tune fast, out-of-tune slow, and noise.

In a pilot study, 21 subjects (who did not take part in the main experiment) evaluated these stimuli according to the experienced arousal and valence on a 5-point Likert scale (ranging from 0 to 4). In-tune music was generally rated as more pleasant than both out-of-tune music and noise (mean valence rating: in-tune fast: 3.04, in-tune slow: 2.5, out-of-tune fast: 1.7, out-of-tune slow: 0.9, noise: 0.6; significant differences between all stimuli). In terms of arousal, the slow musical excerpts were rated as less arousing than the fast excerpts and the noise stimulus (mean arousal rating: in-tune fast: 2.4, in-tune slow: 0.8, out-of-tune fast: 2.14, out-of-tune slow: 1.05, noise: 1.95; significant differences between all stimuli). These five acoustic stimuli were used as background stimuli for the main verbal learning experiment. In this experiment, background stimuli were binaurally presented via headphones (Sennheiser HD 25.1) at approximately 60 dB.

### Verbal memory test

Verbal learning was examined using a standard verbal learning test, which is frequently used for investigations with German-speaking subjects (Verbaler Lerntest, VLT). This test has been shown to validly measure verbal long-term memory [62,63]. The test comprises 160 items and includes neologisms, which evoke either strong (80 items) or weak (80 items) semantic associations. In the test, most of the neologisms are novel (i.e., presented for one time), while 8 of the neologisms are repeatedly presented (7 repetitions), resulting in a total of 104 novel trials and 56 repetition trials. In the procedure used here, subjects were seated in front of a PC screen in an electromagnetically shielded room, and they were asked to discriminate between novel neologisms (NEW) and those that were presented in previous trials (OLD, i.e. repetitions). Subjects were instructed to respond after the presentation of every word by pressing either the right or left button of a computer mouse (right for OLD, left for NEW). Each trial started with a fixation cross (0 - 250 ms) followed by the presentation of a particular word (1150 - 2150 ms). The inter-trial interval, that is, the time between the onsets of the words of two consecutive trials, was 6 seconds. The performance in this memory test was measured using the number of correct responses for recognition of new and old words. For this test we had two parallel versions (version A and B) which allowed for testing the same subjects in two different background conditions (i.e., [background-] and [background+]).

### Psychometrical measures

Several psychological measures were obtained after each experimental condition. First, the participants rated their subjective mood state using the MDBF questionnaire (Multidimensionaler Befindlichkeitsfragebogen) [69]. The MDBF comprises 12 adjectives (content, rested, restless, bad, worn-out, composed, tired, great, uneasy, energetic, relaxed, and unwell) for which the subjects had to indicate on a 5-point scale whether the particular adjective describes their actual feeling (1 = not at all; 5 = perfect). These evaluations are entered into summary scores along the three dimensions valence, arousal, and alertness. The acoustic background stimuli were evaluated using an adapted version of the Music Evaluation Questionnaire (MEQ) [70]. This questionnaire comprises questions evaluating the preference for the presented musical stimuli and how relaxing they are. In this questionnaire, subjects were also asked how they feel after listening to the music (i.e. cheerful, sad, aggressive, harmonious, drowsy, activated, and excited). All items were rated on 5-point Likert scales ranging from (1) not at all to (5) very strongly. The 10 scales were reduced to 3 scales (on the basis of a factor analysis), that is the subjective feeling of pleasantness, activation (arousal), and sadness (sadness).

### EEG recording

The electroencephalogram (EEG) was recorded from 30 scalp electrodes (Ag/AgCl) using a Brain Vision amplifier system (BrainProducts, Germany). Electrodes were placed according to the 10-20 system. Two additional channels were placed below the outer canthi of each eye to record electro-oculograms (EOG). All channels were recorded against a reference electrode located at FCz. EEG and EOG were analogue filtered (0.1-100 Hz) and recorded with a sampling rate of 500 Hz. During recording impedances on all electrodes were kept below 5 k.

### EEG preprocessing

EEG data were preprocessed and analysed by using BrainVision Analyzer (BrainProducts, Munich, Germany) and Matlab (Mathworks, Natick, MA). EEG data were off-line filtered (1-45 Hz), and re-referenced to a common average reference. Artefacts were rejected using an amplitude threshold criterion of ± 100 μV. Independent component analysis was applied to remove ocular artefacts [71,72]. EEG data were then segmented into epochs (-1000 - 4000 ms) relative to the onset of the word stimulus.

**Figure 2:**
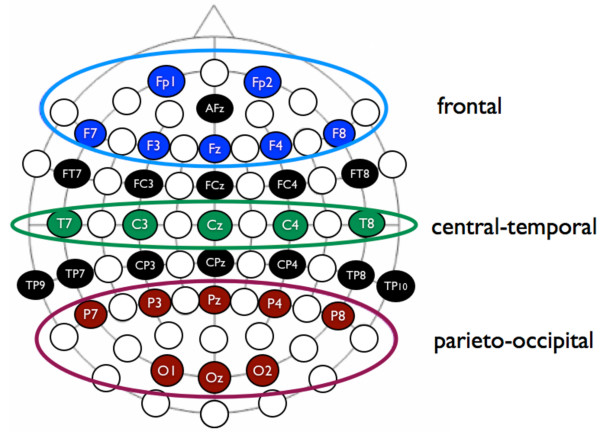
**Schematic description of the definition of the electrode clusters which were used for averaging**.

Analysis of the time course of event-related desynchronization and event-related synchronization was performed according to the classical method described elsewhere [73,74]. We included NEW (i.e., neologisms) and OLD (i.e., presented previously) trials in the event-related synchronization/desynchronization analysis, and only those trials correctly identified as NEW and OLD by the participants. In this study, we calculated event-related synchronization/desynchronization in the alpha band. Several recent combined EEG/fMRI and EEG/PET papers strongly indicate that power in the alpha-band is inversely related to activity in lateral frontal and parietal areas [57-59], and it has been shown that the alpha-band reflects cognitive and memory performance. In the procedure used here, event-related synchronization/desynchronization in the alpha band was analyzed by filtering the artefact-free segments with a digital band-pass filter (8-12 Hz). Amplitude samples were then squared and averaged across all trials, and a low-pass filter (4 Hz) was used to smooth the data. The mean alpha-band activity in latency band -1000 - 0 ms relative to word stimulus onset was defined as intra-experimental resting condition (i.e., the baseline condition). To quantify the power changes during verbal learning, event-related synchronization/desynchronization were calculated according to the following formula: event-related desynchronization (ERD)/event-related synchronization (ERS) = ((band power_task _- band power_baseline_) * 100/band power_baseline_). Note that negative values indicate a relative decrease in the alpha-band (event-related desynchronization) during the experimental condition compared to the baseline condition, while positive values indicate an increase of alpha-band power during the experimental condition (event-related synchronization). In order to avoid multiple comparisons, event-related synchronization/desynchronization values were averaged over 10 time windows with a duration of 400 ms, and were collapsed for the frontal (FP1, FP2, F7, F3, Fz, F4 and F8), central-temporal (T7, C3, Cz, C4 and T8), and parieto-occipital (P7, P3, Pz, P4, P8, O1, Oz and O2) electrode locations [75] (see also Figure 2). Taken together, we obtained a time course of event-related synchronization/desynchronization changes over three different cortical regions (frontal, central-temporal, parieto-occipital), and over a time course of 4000 ms after stimulus presentation (10 event-related synchronization/desynchronization values for the entire time course). For this paper, we restrict our analysis to the first 5 time segments after word presentation, and thus concentrate on a time interval of 0 - 2000 ms after word presentation.

In order to examine possible hemispheric differences, we subsequently calculated left- and right-sided event-related synchronization/desynchronization for the frontal, central-temporal, and parieto-occipital electrodes (left frontal: FP1, F7, F3; right frontal: FP2, F4 and F8; left central-temporal: T7, C3; right-central-temporal: C4 and T8; left parieto-occipital: P7, P3, and O1; right parieto-occipital: P4, P8 and O2). Asymmetric brain activation patterns during music perception have been reported by some studies [76,77]. Different findings have been reported by other studies [78,79].

### Statistical analysis of event-related synchronization/desynchronization

For the main analysis of the event-related synchronization/desynchronization, a four-way ANOVA with the repeated measurements on the following factors was applied: Time Course (5 epochs after word stimulus presentation), Brain Area (3 levels: frontal, central-temporal, parieto-occipital), Background (2 levels: learning with acoustic background = background+, learning without acoustic background = background-), and the grouping factor Group (5 levels: in-tune fast, in-tune-slow, out-of-tune fast, out-of-tune slow, noise). Following this, we computed a four-way repeated measurements ANOVA, including the event-related synchronization/desynchronization data obtained for the left- and right-sided electrodes of interest, to examine whether hemispheric differences might influence the overall effect. For this, we used the multivariate approach to handle with the problem of heteroscedasticity [80]. Results were considered as significant at the level of p < 0.01. We used this more conservative approach in order to guard against problems associated with multiple testing. All statistical analyses were performed using the statistical software package SPSS 17.01 (MAC version). In case of significant interaction effects post-hoc paired t-tests were computed using the Bonferroni-Holm correction [81].

In order to assess whether there are between hemispheric differences in the cortical activations during listening to background music, we subjected the event-related synchronization/desynchronization data of the frontal, central-temporal, and parietal-occipital ROIs separately to a four-way ANOVA with Hemisphere (left vs. right), Group, Background, and Time as factors (Hemisphere, Group, and Background are repeated measurements factors). If background music and especially background music of different valence would evoke different lateralization patterns than the interaction between Hemisphere, Group or Hemisphere, Group, and Background should become significant. Thus, we were only interested in these interactions.

## Results

### Learning performance

**Figure 3:**
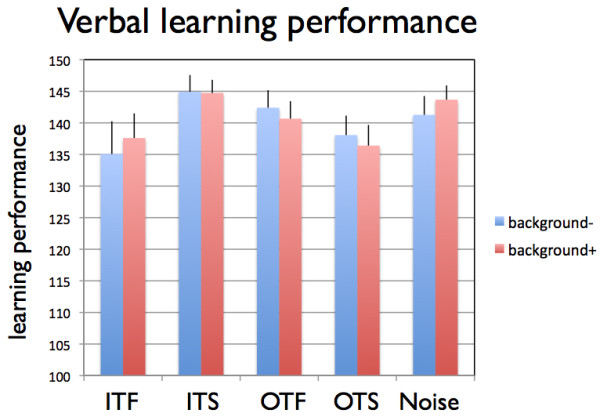
**Mean verbal learning performance (number of correct responses) for the five experimental groups broken down for learning with (background+ in red) and without (background- in blue) musical background music**. ITF: in-tune fast, ITS: in-tune slow, OTF: out-of-tune fast, OTS: out-of-tune slow. Shown are means and standard errors (as vertical bars).

Subjecting the verbal learning data to a 2-way repeated measurements ANOVA with repeated measurements on one factor (Background: background+ and background-) and the grouping factor (Group: in-tune fast, in-tune slow, out-of-tune fast, out-of-tune slow, noise) revealed no significant main effect (Background: F(1,70) = 0.073, p = 0.788, eta^2 ^= 0.001; Group: F(4,70) = 1.42,p = 0.235, eta^2 ^= 0.075) nor a significant interaction (F(4,70) = 0.90, p = 0.47, eta^2 ^= 0.049) (Figure 3 shows the means and standard errors).

### Emotional evaluation of acoustic background

The valence and arousal ratings for the three background conditions were subjected to separate repeated measurements ANOVAs. The valence measures were significantly different for the five background conditions (F(4,70) = 4.75, p < 0.002). Subsequently performed Bonferroni corrected t-tests revealed significant differences between the in-tune and out-of-tune conditions (mean valence rating ± standard deviation: ITF = 3.5 ± 1.1; ITS = 3.1 ± 0.7; OTF = 2.7 ± 1.0; OTS = 2.3 ± 0.9; Noise = 2.2 ± 1.1). For the arousal rating we obtained no significant difference between the five conditions (F(4,70) = 1.07, p = 0.378).

The MEQ rating data were subjected to three 2-way ANOVAs with one repeated measurements factor (Background: background+ vs. background-) and the grouping factor (Group). There was a significant main effect for Group with respect to pleasantness, with the in-tune melodies receiving the highest pleasantness ratings and the noise stimulus the lowest (F(4,70) = 12.5, p < 0.001 eta^2 ^= 0.42). There was also a trend for interaction between Background and Group (F(4,70) = 2.40, p = 0.06, eta^2 ^= 0.12), which is qualified by reduced pleasantness ratings for the in-tune melodies for the condition in which the subjects were learning. For the sadness scale we obtained a main effect for Background (F(1,70) = 8.14, p = 0.006, eta^2 ^= 0.10) qualified by lower sadness ratings for the music heard while the subjects were learning.

The kind of acoustic background also influenced the subjective experience of pleasantness (F(1,70) = 24.6, p < 0.001, eta^2 ^= 0.26), with less pleasantness during learning while acoustic background stimulation was present. The subjective feeling of arousal and sadness did not change as a function of different acoustic background conditions.

### EEG data

**Figure 4:**
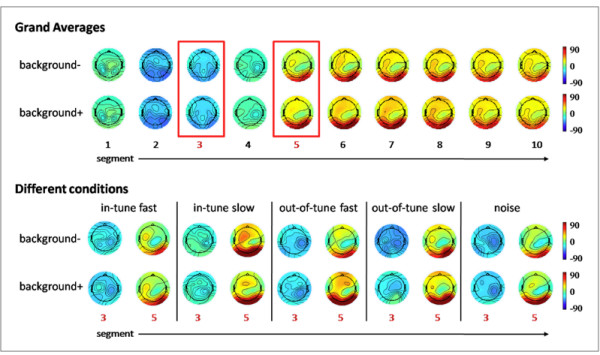
**Mean ERDs and ERSs (in %) as topoplots broken down for the 10 time segments and for learning with (background+) and without (background-) musical background music**. Blue indicates ERD and red ERS. The time segments are printed under the topoplots (upper panel). In the lower panel the topoplots are shown broken down for the two most interesting time segments (3 and 5) and the different musical background conditions.

**Figure 5:**
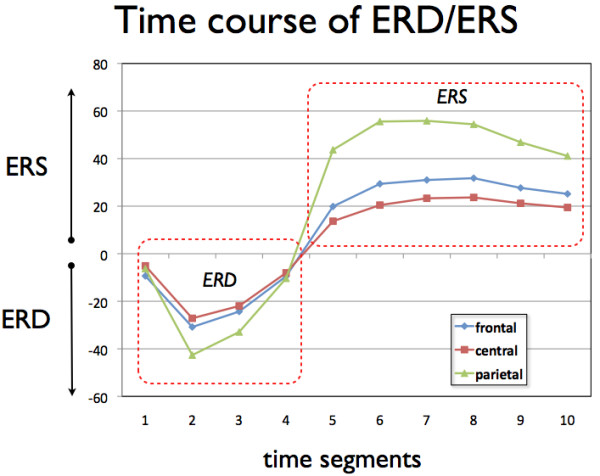
**Time courses of the changes in alpha-band power after word presentation broken down for the three brain regions of interest**. Each time segment represents the mean ERD/ERS over a 400 ms segment (averaged across the different groups and the 2 background conditions). Indicated are the 10 time segments after word presentation. Negative values indicate a decrease in alpha-band power (event related desynchronization: ERD) while positive values indicate an increase of alpha-band power (event related synchronization: ERS) during learning compared to the baseline condition.

The event-related synchronization/desynchronization data of the alpha band were first subjected to a 4-way ANOVA with repeated measurements on three factors (Time = 5 levels; Brain Area = 3 levels: frontal, central-temporal, and parieto-occipital; Background = 2 levels: background+ and background-) and one grouping factor (Group; 5 levels: in-tune fast, in-tune slow, out-of-tune fast, out-of-tune slow, noise). Where possible we used the multivariate approach to test the within-subjects effects, since this test is robust against violations of heteroscedasticity [80]. Figure 4 demonstrates the mean ERDs and ERSs as topoplots broken down for the 10 time segments and for learning with (background+) and without (background-) musical background. Figure 5 depicts the grand averages of event-related synchronization and desynchronization for the frontal, central-temporal, and parieto-occipital leads.

**Figure 6:**
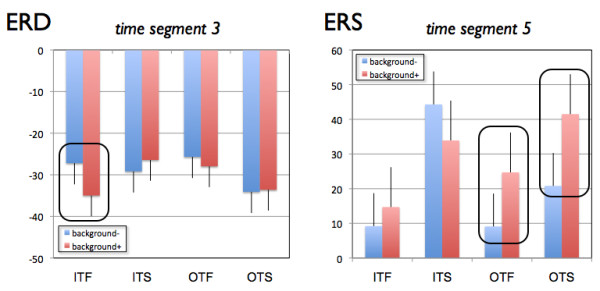
**Means and standard errors for event related desynchronization (ERD): (A) and event related synchronization (ERS): (B) values at time segment 3 and 5 (800 - 1200 ms and 1600 - 2000 ms after word presentation, respectively)**. **A) **Mean ERD for the five experimental groups broken down for learning with (background+ in red) and without (background- in blue) musical background music. **B) **Mean ERS for the five experimental groups broken down for learning with (background+ in red) and without (background- in blue) musical background music. ITF: in-tune fast, ITS: in-tune slow, OTF: out-of-tune fast, OTS: out-of-tune slow).

This complex ANOVA revealed several main effects and interactions. The event-related synchronization/desynchronization data showed a typical time course with a strong event-related desynchronization peaking at the second time segment (400 - 800 ms after word presentation). After reaching the maximum event-related desynchronization, the alpha-band synchronises again with the strongest event-related synchronization at the 5th time segment (1600 - 2000 ms after word presentation). This time course is highly significant (F(4,67) = 106.2, p < 0.001, eta^2 ^= 0.86). The time courses of event-related synchronization/desynchronization are different for the different brain areas with larger event-related desynchronization for the parieto-occipital leads at the second time segment and larger event-related synchronization also at the parieto-occipital leads for the 5th time segment after word presentation (F(8, 63) = 34.50, p < 0001, eta^2 ^= 0.81) (Figure 5). There was also a significant Background × Time × Group interaction (F(16,280) = 2.27, p = 0.004, eta^2 ^= 0.11). In order to delineate this three-way interaction we conducted two-way ANOVAs with Background and Group as factors separately for each time segment. There were only significant Background × Group interactions for the time segments 3 and 5 (T3: F(4,70) = 2.6, p = 0.038, eta^2 ^= 0.13; T5: F(4,70) = 2.7, p = 0.036, eta^2 ^= 0.13). The interaction at time segment 3 (800 - 1200 ms after word presentation) was qualified by a larger event-related desynchronization for verbal learning with background music (background+) for the in-tune-fast group (F(1,14) = 9.2, p = 0.009, eta^2 ^= 0.397 significant after Bonferroni-Holm correction at the level of p = 0.05). The interaction at time segment 5 (1600 - 2000 ms after word presentation) was qualified by larger event-related synchronization for the out-of-tune-fast group during background+ (F(1,14) = 6.3, p = 0.025, eta^2 ^= 0.13, marginally significant after Bonferroni-Holm correction at the level of p = 0.05). Figure 6 shows the mean event-related synchronization/desynchronization for the time segments 3 and 5.

The ANOVA analysis conducted to examine potential between-hemisphere differences in terms of event-related synchronization/desynchronization revealed that none of the interactions of interest (Hemisphere × Group, Hemisphere × Group × Background) turned out to be significant, even if the statistical threshold was lowered to p = 0.10.

## Discussion

The present study examined the impact of background music on verbal learning performance. For this, we presented musical excerpts that were systematically varied in consonance and tempo. According to the *Arousal-Emotion hypothesis*, we anticipated that consonant and arousing musical excerpts would influence verbal learning positively, while dissonant musical excerpts would have a detrimental effect on learning. Drawing on the theory of *changing state effects*, we anticipated that rapidly changing auditory information would distract verbal learning more seriously than slowly changing music. Thus, slower musical pieces were expected to exert less detrimental effects on verbal learning than faster music. In order to control for the global influence of familiarity with and preference for specific music styles, we designed novel in-house musical excerpts that were unknown to the subjects. Using these excerpts, we did not uncover any substantial and consistent influence of background music on verbal learning.

The effect of passive music listening on cognitive performance is a long-standing matter of research. The findings of studies on the effects of background music on cognitive tasks are highly inconsistent, reporting either no effect, declines or improvements in performance (see the relevant literature mentioned in the introduction). The difference between the studies published to date and the present study is that we used novel musical excerpts and applied experimental conditions controlling for different levels of tempo and consonance. According to the *Arousal-Emotion *hypothesis, we hypothesised that positive background music arouses the perceiver and evokes positive affect. However, this hypothesis was not supported by our data since there was no beneficial effect of positive background music on verbal learning. In addition, according to the theory of *changing state effects *we anticipated that rapidly changing auditory information would distract verbal learning more seriously than slowly changing music. This theory also was not supported by our data.

As mentioned above, the findings of previously published studies examining the influence of background music on verbal learning and other cognitive processes are inconsistent, with more studies reporting no influence on verbal learning and other cognitive tasks. But before we argue too strongly about non-existing effects of background music on verbal learning we will discuss the differences between our study and the previous studies in this research area. Firstly, it is possible that the musical excerpts used may not have induced sufficiently strong arousal and emotional feelings to exert beneficial or detrimental effects on verbal learning. Although the four different musical excerpts significantly differed in terms of valence and arousal, the differences were a little smaller in this experiment than in the pilot experiment. Had we used musical excerpts that induce stronger emotional and arousal reactions, the effect on verbal learning may have been stronger. In addition, the difference between the slow and fast music in terms of changing auditory cues might not have been strong enough to influence verbal learning. There is however no available data to date to indicate "optimal" or more "optimal" levels of arousal and/or emotion and of changes in auditory cues for facilitating verbal learning.

A further aspect that distinguishes our study from previous studies is that the musical pieces were unknown to the subjects. It has been shown that music has an important role in autobiographical memory formation such that familiar and personally enjoyable and arousing music can elicit or more easily facilitate the retrieval of autobiographical memories (and possible other memory aspects) [48-50]. It is conceivable that the entire memory system (not only the autobiographical memory) is activated (aroused) by this kind of music, which in turn improves encoding and recall of information. Recently, Särkämo et al. [82] demonstrated that listening to preferred music improved verbal memory and attentional performance in stroke patients, thus supporting the *Arousal-Emotion *hypothesis. However, the specific mechanisms responsible for improving memory functions while listening to music (if indeed present) are still unclear. It is conceivable that the unknown musical pieces used in our study did not activate the memory system, thus, exerting no influence on the verbal learning system.

Although verbal learning performance did not differ between the different background stimulation conditions, there were some interesting differences in the underlying cortical activations. Before discussing them, we will outline the similarities in cortical activations observed for different background conditions. During learning, there was a general increase of event-related desynchronization 400-1200 ms after word presentation followed by an event-related synchronization in a fronto-parietal network. This time course indicates that this network is cortically more activated during encoding and retrieval in the first 1200 ms. After this, the activation pattern changes to event-related synchronization, which most likely reflects top-down inhibition processes supporting the consolidation of learned material [56].

Although the general pattern of cortical activation was quite similar across the different background conditions, we also identified some considerable differences. In the time window between 800 - 1200 ms after word presentation, we found stronger event-related desynchronization at frontal and parietal-occipital areas for the in-tune-fast group only. Clearly, frontal and parieto-occipital areas are stronger activated during verbal learning in the ambient setting of in-tune-fast music but not in the other conditions. Frontal and parietal areas are strongly involved in different stages of learning. The frontal cortex is involved in encoding and retrieval of information, while the parieto-occipital regions are part of a network involved in storing information [83-87]. One of the reasons for this event-related desynchronization increase could be that the fronto-parietal network devotes greater cortical resources to verbal learning material in the context of in-tune fast music (which by the way is the musical piece rated as being most pleasant and arousing). But we did not find a corresponding behavioural difference in learning performance. Thus, the activation increase may have been too small to be reflected in a behaviour-enhancing effect. A further possibility is that the in-tune-fast music is the most distracting of the background music types, therefore eliciting more bottom-up driven attention compared with the other musical pieces and constraining attentional resource availability for the verbal material. The lack of a decline in learning performance suggests that attentional resource capacity was such that this distracting effect (if indeed present) was compensated for.

A further finding is the stronger event-related synchronization in the fronto-parietal network at time segment 5 (1600 - 2000 ms after word presentation) for the out-of-tune groups and especially for the out-of-tune-fast group. Event-related synchronization is considered to reflect the action of inhibitory processes after a phase of cortical activation. For example, Klimesch et al. [56] propose that event-related synchronization reflects a kind of top-down inhibitory influence. Presumably, the subjects exert greater top-down inhibitory control to overcome the adverse impact on learning in the context of listening to out-of-tune background music.

We did not find different lateralisation patterns for event-related synchronization/desynchronization in the context of the different background music conditions. This is in contrast to some EEG studies reporting between hemispheric differences in terms of neurophysiological activation during listening of music of different valence. Two studies identified left-sided increase of activation especially when the subjects listened to positively valenced music opposed to negative music evoking a preponderance of activation on the right hemisphere (mostly in the frontal cortex) [76,77,88]. These findings are in correspondence with studies demonstrating dominance of left-sided activation during approach-related behaviour and stronger activation on the right during avoidance-related behaviour [89]. Thus, music eliciting positive emotions should also evoke more left-sided activation (especially in the frontal cortex), but not all studies support these assumptions. For example, the studies of Baumgartner et al. [78] and Sammler et al. [79] did not uncover lateralised activations in terms of Alpha power changes during listening to positive music. Sammler et al. identified an increase in midline Theta activity and not a lateralised activation pattern. In addition, most fMRI studies measuring cortical and subcortical activation patterns during music listening did not report lateralised brain activations [90-95]. All of these studies report mainly bilateral activation in the limbic system. One study also demonstrates that the brain responses to emotional music substantially changes over time [96]. In fact, a differentially lateralised activation pattern due to the valence of the presented music is not a typical finding. However, we believe that the particular pattern of brain activation and possible lateralisation patterns are due to several additional factors influencing brain activation during music listening. For example, experience or preference for particular music are potential candidates as modulatory factors. Which of these factors are indeed responsible for lateralised activations can not be clarified on the basis of current knowledge.

Future experiments should use musical pieces with which subjects are familiar and which evoke strong emotional feelings. Such musical pieces may influence memory performance and the associated cortical activations entirely differently. Future experiments should also seek to examine systematically the effect of pre-experimentally present or experimentally induced personal belief in the ability of background music to enhance performance and, depending on the findings, this should be controlled for in other subsequent studies. There may also be as yet undocumented, strong inter-individual differences in the modulatory impact of music on various psychological functions. Interestingly, subjects have different attention and empathy performance styles, and this may influence the performance of different cognitive functions.

## Limitations

A methodological limitation of this study is the use of artificial musical stimuli, which are unknown to the subjects. In general we listen to music we really like when we have the opportunity to deliberately chose music. Thus, it might be that in case of listening to music we really appreciate to listen to while we learn the results might be entirely different. However, this has to be shown in future experiments.

## Conclusion

Using different background music varying in tempo and consonance, we found no influence of background music on verbal learning. There were only changes in cortical activation in a fronto-parietal network (as measured with event-related desynchronization) around 400 - 800 ms after word presentation for the in-tune-fast-group, most likely reflecting a larger recruitment of cortical resources devoted to the control of memory processes. For the out-of-tune groups we found stronger event-related synchronization around 1600 - 2000 ms in a fronto-parietal network after word presentation, this thought to reflect stronger top-down inhibitory influences on the memory system. We suggest that this top-down inhibitory influence is at least in part a response to the slightly more distracting out-of-tune music that enables the memory system to adequately reengage in processing the verbal material.

## Competing interests

The authors declare that they have no competing interests.

## Authors' contributions

LJ and PS both designed the experimental paradigm, performed the statistical analysis and drafted the manuscript. All authors read and approved the final manuscript.
