# Correction: Music listening while you learn: No influence of background music on verbal learning

**DOI:** 10.1186/1744-9081-6-11

**Published:** 2010-02-08

**Authors:** Lutz Jäncke, Pascale Sandmann

**Affiliations:** 1 University of Zurich, Psychological Institute, Department of Neuropsychology, Switzerland

## Correction

After publication of this work [1], we noted that there was some missing data in Figure six (Figure 1). Extra columns to show the control values of 'Noise' have been added, and the correct figure and figure legend have been included here.

**Figure 1:**
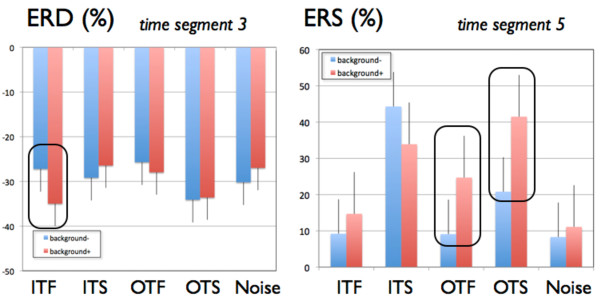
**Means and standard errors for event related desynchronization (ERD) (left) and event-related synchronization (ERS) (right)**. The figure shows ERD/ERS values (%) at time segments 3 and 5 (800 - 1200 ms and 1600 - 2000 ms after word presentation). Left: Mean ERD for the five experimental groups broken down for learning with (background+ in red) and without (background- in blue) background music. Right: Mean ERS for the five experimental groups broken down for learning with (background+ in red) and without (background- in blue) background music. ITF: in-tune fast, ITS: in-tune slow, OTF: out-of-tune fast, OTS: out-of-tune slow, Noise: noise condition).
